# The preload force affects the perception threshold of muscle vibration-induced movement illusions

**DOI:** 10.1007/s00221-018-5402-4

**Published:** 2018-10-19

**Authors:** Francesca Ferrari, Francesco Clemente, Christian Cipriani

**Affiliations:** The BioRobotics Institute, Scuola Superiore Sant’Anna, Viale Rinaldo Piaggio, 34, 56025 Pontedera, PI Italy

**Keywords:** Proprioception, Muscle spindles, Vibration, Sensory feedback, Psychophysics, Perception threshold

## Abstract

The control and the execution of motor tasks are largely influenced by proprioceptive feedback, i.e. the information about the position and movement of the body. In 1972, it was discovered that a vibratory stimulation applied non-invasively to a muscle or a tendon induces a movement illusion consistent with the elongation of the vibrated muscle/tendon. Although this phenomenon was reported by several studies, it is still unclear how to reliably reproduce it because of the many different features of the stimulation altering the sensation (e.g. frequency, duration, location). By performing a psychophysical test, we analysed the effects of the stimulation point and the preload force on the minimum stimulation amplitude needed to elicit an illusion of movement. In particular, we stimulated two groups of healthy subjects on three target regions of the biceps brachii muscle (the distal tendon, the muscle belly and one of the proximal tendons) applying three preload force ranges (0.5–0.75N, 1–2N and 3–4N). Our results showed that the minimum stimulation amplitude eliciting a sensation is affected by the preload force. On the contrary, it did not change significantly among the three stimulated regions. Nevertheless, the reported vividness of the illusion of movement changed across the stimulated points decreasing while moving from the distal to the proximal tendons. Overall, these outcomes contribute to the scientific debate on the features that modulate the vibration-induced movement illusion proposing ways to increase the reliability of the procedure in basic and applied research studies.

## Introduction

Proprioception refers to the ability to sense the position and movement of one’s own body (Sherrington 1906). This sensation is mediated by several biological receptors: mechanoreceptors in the skin, joint capsule receptors, Golgi tendon organs between skeletal muscles and tendons, and spindles within the skeletal muscles belly (Kandel 2013). These receptors provide proprioceptive sensory feedback to the central nervous system, which uses it to refine balance and movements. Indeed, in their absence, movements are clumsy, poorly coordinated and inadequately adapted to complex tasks (Gordon et al. 1995; Kandel 2013). Albeit it is known that such receptors are sensitive to skin stretch around the joints (Collins et al. 2005) and to changes of muscle length and tension, the specific role of each class of receptors in encoding proprioceptive information is still a matter of debate (Marasco et al. 2017).

Nonetheless, several studies showed that it is possible to artificially elicit an illusion of movement (IoM) by mechanically vibrating the tendons or the muscles non-invasively, through the skin (Roll et al. 1989; Albert et al. 2006; White and Proske 2009). In particular, in their pioneering experiment, Goodwin et al. (1972a, b) applied a 100 Hz mechanical vibration, non-invasively, above the distal tendons of both the biceps brachii and the triceps brachii of one arm of the participants. At the same time, they were asked to track the perceived position of the vibrated arm using the contralateral one. Their results showed a misalignment between the positions of the two arms which was always going towards the elongation of the stimulated muscle. Following studies suggested that the illusion is probably mediated by the activation of muscle spindles (Roll and Vedel 1982). Indeed, these sensors were found to respond to similar mechanical stimulations in studies involving animals (Brown et al. 1967) or humans (Burke et al. 1976; Roll and Vedel 1982).

Since its first demonstration, the vibration-induced movement illusion has been widely investigated (Jones 1988; Calvin-Figuière et al. 1999; White and Proske 2009), and used in several neurophysiological paradigms to study the mechanisms involved in the human sensory motor system (Roll et al. 1989; Naito et al. 1999, 2016; Fallon and Macefield 2007). Albeit this is now a well-known phenomenon, how to induce repeatable movement (kinaesthetic) sensations using vibration is still unclear. Indeed, the quality of the sensation was found to depend on several parameters (Taylor et al. 2017): the stimulation point, the preload force (PF) applied on it by the vibrator and the features of the mechanical vibration, e.g. its frequency, duration and peak-to-peak displacement (i.e. the amplitude).

Among these features, the vibration frequency and duration, being the most reliably controllable, were deeply investigated. It is thus well known that stronger illusions are elicited with mechanical vibrations in the 70–100 Hz range (Roll et al. 1989; Naito et al. 1999; Albert et al. 2006), and lasting from a few (e.g. 1–3 s) (Goodwin et al. 1972b; Roll and Vedel 1982), to several seconds (e.g. 15–30 s) (Cordo et al. 2005; Fuentes et al. 2012; Tidoni et al. 2015). Regarding the stimulation amplitude and the PF, only a few studies assessed their influence on the quality of the sensation. Concerning the former, Schofield and colleagues reported that by vibrating the regions between the muscles and the tendons of the biceps and triceps brachii the strength of the IoM, the range of motion and velocity of the perceived movement positively correlate with the stimulation amplitude (Schofield et al. 2015).

On the other hand, the effects of the PF were rarely investigated. An important contribution was presented by Cordo and colleagues (Cordo et al. 1993), who measured through microneurography the response of the sensory afferents while vibrating—non-invasively—the tendons of the tibialis anterior muscle. In particular, they were able to precisely control the applied PF as well as the amplitude of the vibration, by means of a servomechanism that implemented a force feedback loop. Their results proved a positive correlation between afferents discharge and applied PF. However, they did not report whether and how the IoM was altered during their experiment. To the best of our knowledge, only one study using psychophysics methods investigated the effects of PF on the quality of the IoM through questionnaires (Fusco et al. 2016). Specifically, the authors tested two PFs (2.4 N and 4.2 N) and found that when the larger PF was used, the vividness, the range of motion and the duration of the IoM increased as well.

While most of the literature focused on stimulating the distal tendons (Roll et al. 1989; Albert et al. 2006; Tidoni et al. 2015), only few studies reported that it is possible to elicit an IoM by vibrating the muscle belly (Ansems et al. 2006; White and Proske 2009), and the proximal tendon (Craske 1977). However, to the best of our knowledge, a systematic analysis of the effects of the stimulation point on the IoM was never carried out. Hence, it is still unknown whether and how the elicited movement illusions are affected when stimulating different areas of the muscle.

To fill these gaps, in this study we investigated how the stimulation point and the PF affected the minimum stimulation amplitude needed to elicit an illusion of movement. In particular, three different regions (the distal tendon, the muscle belly and one of the proximal tendons) of the biceps brachii muscle were stimulated and, for each point, two PF ranges were tested (1–2N, 3–4N). We evaluated the effects of these parameters by means of an adaptive non-parametric psychophysical test on 16 healthy participants. Our results show that the PF significantly affects the minimum stimulation amplitude needed to elicit an illusion. On the contrary, no effects were observed by changing the stimulation point. For this reason, the effects of the stimulation point were further investigated by including an additional, lower, PF range (0.5–0.75N). This supplementary experiment was included to understand whether the larger PF was masking the effects of the stimulation point on the IoM. However, this was not the case, confirming no effects of the stimulation point on the minimum stimulation amplitude needed to elicit an illusion.

Our outcomes clearly show that the PF has to be appropriately controlled to produce a consistent kinaesthetic illusion and to allow for a fair comparison between studies and encourage further investigation into the effects of different stimulation parameters in eliciting an IoM.

## Materials and methods

**Fig. 1:**
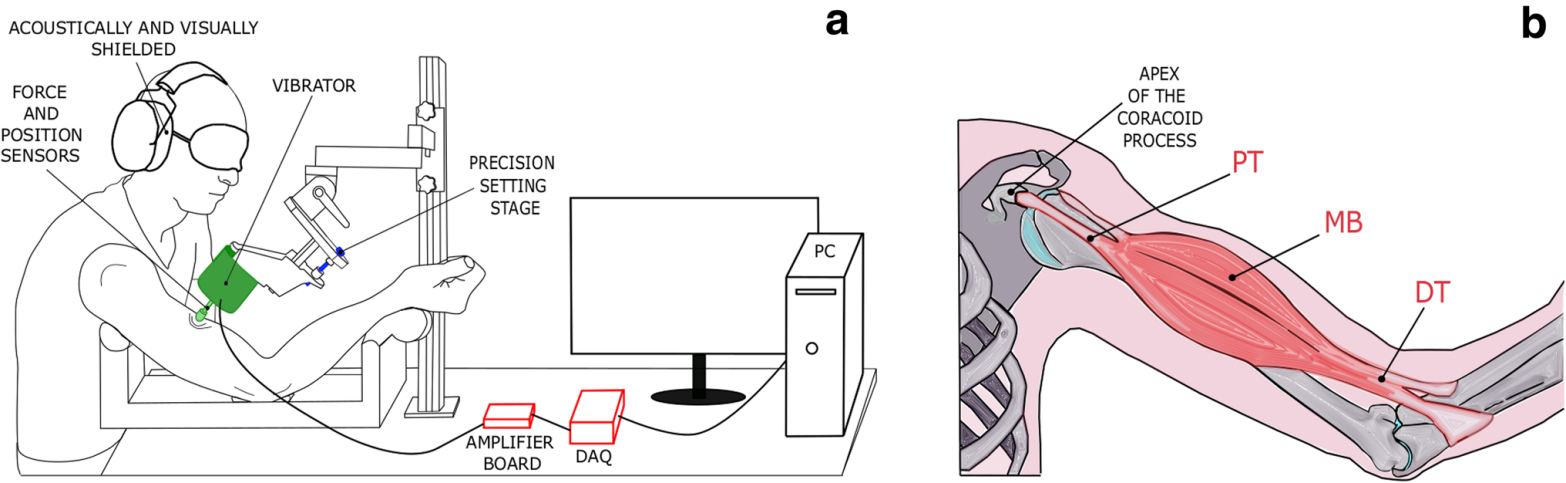
Experimental setup. **a** The setup comprised a PC, a data acquisition board (DAQ), a signal amplifier board and a vibrator. The vibrator was equipped with position and force sensors. The subjects were seated with the elbow angle at around 120°. They were wearing headphones (to avoid distraction due to the noise produced by the vibrator) and were blindfolded (to avoid incongruent visual feedback of the arm, which can disrupt the illusion (Guerraz et al. 2012)). The precision setting stage supporting the vibrator guaranteed a fine control of the applied preload force. **b** Location of the stimulated points (in red). DT indicates the distal tendon, MB the muscle belly and PT the proximal tendon. The apex of the coracoid process was used as an anatomical landmark to localize the PT

The experimental setup comprised a PC, a data acquisition board (DAQ, USB-6211, National Instrument Corp.), a signal amplifier board (TA2024, Sure Electronics) and a vibrator (Mini Shaker 4810, Brüel & Kjær). The tip of the vibrator (10 mm diameter, hemispherical) was equipped with a Hall-effect analog sensor (SS490 Series, Honeywell Inc.) and a uniaxial load cell (Model S215, Strain Measurement Device) to measure its position and the force it applied on the skin, respectively (Fig. 1a). The vibrator was supported by an adjustable mechanical arm and a manual precision setting stage that allowed the experimenter to finely adjust its position with respect to the participants’ arm. To characterize the properties of the vibrator interfacing with the body tissues, the probe displacement was measured without any load and when pressing on the skin at the different PF levels used during the experiment (see protocol).

The PC ran a custom LabVIEW (National Instruments Corp.) application, which monitored the vibrator tip force and position, controlled the vibratory stimulation and implemented the experimental protocol. In particular, through the DAQ, the application generated a sinusoidal excitation signal that, after being amplified, was converted into a mechanical vibration by the transducer and used to stimulate the participants. Each excitation signal was generated at a frequency of 80 Hz, lasted for 2 s (rising/falling time of 300/400 ms) and had a peak-to-peak amplitude dependent on the experiment phase (see below).

Sixteen healthy adults (age 25–35, nine females, all right-handed) with no known history of neurological disorders participated in the experiment. Informed consent in accordance with the Declaration of Helsinki was obtained before conducting the experiments from each subject. This study was approved by the local ethical committee of the Scuola Superiore Sant’Anna, Pisa, Italy (request no. 6/2017). The methods were carried out in accordance with the approved guidelines.

### Protocol

The experiment aimed at measuring the minimum stimulation amplitude (i.e. the amplitude threshold, AT) eliciting an illusion of movement in the subjects. The stimulation amplitude was defined as the peak-to-peak displacement (in mm) of the vibrator tip.

Each participant sat on a chair with the right arm supported by a stand, which constrained the elbow to bend at 120° (Fig. 1a). It was shown earlier that the specific arm posture does not affect the strength of the illusion (Schofield et al. 2015) and the elbow bent at 120° allowed for a comfortable posture throughout the experimental session. The participant was asked to keep the arm relaxed and to maintain a stable posture during the experiment. The biceps brachii was stimulated in three regions: the distal tendon (DT—close to the insertion of the muscle), the muscle belly (MB), and the proximal tendon (PT). The DT, attached to the radial tuberosity, was easily localized through palpation at the level of the elbow. To identify the MB, the subjects were asked to bend their elbow at 90° and perform an isometric contraction. In this configuration, the MB was identified as the central point on the skin lying on the plane of maximum cross-sectional area of the arm. Similarly, the PT was identified through palpation, as the point ~ 30 mm distally from the origin of the short head of the biceps, i.e. the apex of the coracoid process of the scapula—(Fig. 1b). In particular, while the experimenter kept his fingers in that specific point the participant was asked to flex/extend the forearm. The muscle movement, perceived through the skin surface, helped the experimenter to unequivocally identify the biceps tendon. In some cases when the experimenter had difficulties in localizing the tendon, an alternative procedure was used. First, the belly of the muscle was identified as previously explained. From this point, while the participant flexed/extended the elbow, the experimenter tracked through palpation the perceived movement until the origin of the muscle. Again, the PT was selected around ~ 30 mm distally from the origin.

While participants were aware that they would receive a stimulation that could induce an IoM, they received no information about neither the joints involved in the movement nor its direction (i.e. flexion or extension). For this reason, to make them quickly aware of the illusion, the DT was chosen as the first stimulation point for all subjects. Indeed, from pilot experiments, we learned that this point was quickly identifiable by the experimenter and that the subjects became aware of the sensation more rapidly. MB and PT were stimulated afterwards, being the order randomized and counterbalanced across participants.

Two different stimulation phases were conducted for each stimulation point: a training phase and an experimental phase. The training phase consisted of one to five cycles of five consecutive stimulations. In this phase, the amplitude of the stimulation was 1 mm. At the end of each cycle, the participant verbally reported if he/she perceived any IoM. The training phase helped participants in getting accustomed to the expected illusion, i.e., the extension of the elbow, it is indeed known that some people do not perceive any illusion or take time to become aware of it (Taylor et al. 2017). In the case that participants were able to perceive the IoM during the training phase, the experimental phase was carried out; otherwise, the experiment was stopped.

During the experimental phase, each target point was tested twice, consecutively, with a lower PF (PF_L_) ranging between 1 N and 2 N and with a higher PF (PF_H_) ranging between 3 N and 4 N. This accounted for a total of six experimental conditions (3 stimulation points × 2 PF ranges). The order of application of these PF ranges was randomized across stimulating points and participants. Before each trial, the actual PF as measured by the load cell was manually adjusted by the experimenter using the precision stage, to ensure that it fell within the desired range.

**Fig. 2:**
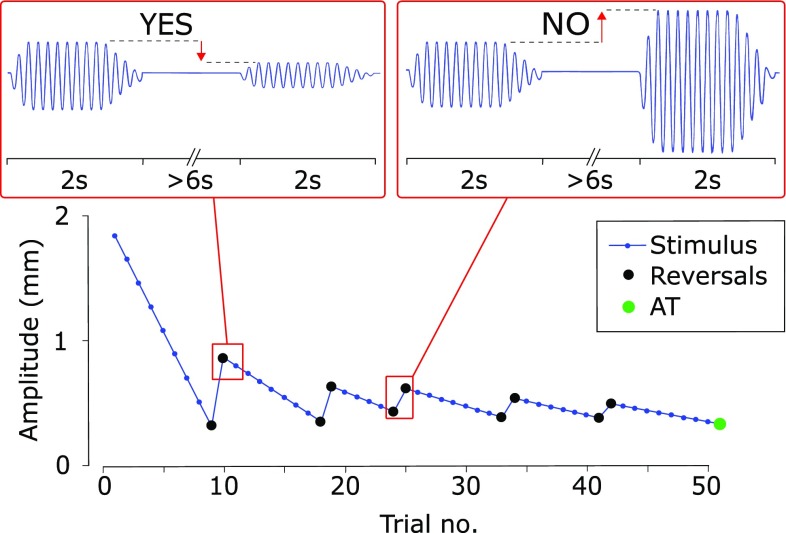
Experiment trials. For each experimental condition (3 stimulated points × 2 PF ranges = 6 conditions for the first experiment; 3 stimulated points × 1 PF range = 3 conditions for the second experiment), the subjects received 50 stimulation trials. The experimenter changed the amplitude of the stimulation according to a stochastic approximation staircase (SAS, see text) targeting 85% detection threshold. In particular, the vibration amplitude decreased if the subject perceived an illusion of movement (top left box), otherwise it increased (top right box). The amplitude threshold (AT) was chosen as the stimulation amplitude that would have been presented on the 51st stimulation. The stimulation lasted for 2 s and a pause between 6 and 20 s separated two consecutive stimulations to allow the subjects to report on the presence of the IoM

To determine the AT, a standard yes/no adaptive threshold procedure was used (Kingdom and Prins 2010). In particular, a series of vibratory stimulations was presented to the subject; on each trial (i.e. for each vibration) the subject was asked to respond ‘Yes’ (or ‘No’) if he/she perceived (or not) a clear IoM. If he/she responded ‘Yes’, in the next trial the amplitude of the stimulation decreased, otherwise it increased (Fig. 2). A stochastic approximation staircase (SAS) was used to calculate the amplitude of the stimulation, according to the following equation (Kesten 1958):

1. $${x_{n+1}}={x_n} - \frac{c}{{1+m}}\left( {{Z_n} - \phi } \right)$$

where ${x_n}$ is the amplitude of the stimulation during the previous trial; ${Z_n}$ is set to 1 if the participant perceived an IoM (i.e. responded ‘Yes’) or 0 if he/she did not report any sensation (i.e. responded ‘No’); $m$ is the number of reversals (how many times from the first trial of the staircase to the current one, the answer, ${Z_n}$, switches from ‘Yes’ to ‘No’ and vice versa); $\phi$ is the target threshold probability (set to 0.85); $c$ is a suitable constant (chosen through pilot experiments to be 0.4).

To set the starting stimulation amplitude of the SAS, the method of limits was used (Kingdom and Prins 2010) for each stimulation point and each subject. In particular, three ascending and three descending series were performed, and the resulting threshold was averaged among the six series. The amplitude of the first stimulation (*x*_1_) was thus set clearly suprathreshold in a way that it ensured at least five trials before reaching the rough estimate, to guarantee proper efficiency of the staircase (Faes et al. 2007).

A pause between 6 and 20 s separated two consecutive stimulations to allow the subjects to report their answer and to avoid temporal overlap between any after-effect sensation and the consecutive stimulation (Kito et al. 2006). We followed the recommendations and constraints indicated by Faes and colleagues to ensure an accurate estimation of the AT (Faes et al. 2007). In particular, the SAS was terminated after 50 trials, and the value of the stimulation that would have been presented as the 51st trial was chosen as the AT (Clemente et al. 2017).

The collected data were analysed using a two-way repeated-measure analysis of variance (RM-ANOVA) to evaluate the influence of the PF and stimulation point on the AT. Statistical significance in the RM-ANOVA was followed by post hoc pairwise comparisons with Bonferroni correction.

As the analysis showed no statistical difference in the AT between the three stimulation points, an additional PF range (0.5–0.75 N—namely PF_LL_) was tested on 12 additional subjects following the same protocol described above, to assess differences across the three stimulation points (3 stimulation points × 1 PF range). These subjects had prior experience with IoM, and unlike the previous case, the presentation order of the three stimulation points was randomized across the subjects. A one-way RM-ANOVA was used to compare the AT across the stimulation points. The subjects were also asked to rank the three stimulated points based on the vividness of the perceived illusion by reporting which they considered the first, the second and third most vivid stimulation point. A Friedman test was used to compare the vividness ranking across the three points and a Wilcoxon signed-rank test with Bonferroni correction was used for pairwise comparisons.

The Kolmogorov–Smirnov test was used to verify normality in the parametric tests, and in all cases, a *p* value < 0.05 was considered for statistical significance.

## Results

In the first part of this section, we present the mechanical characterization of the vibrator (i.e. the relation between the PF and the vibration amplitude) while it is in contact with the skin. Subsequently, we qualitatively report on the ability of the subjects in experiencing an IoM and, finally, we report the ATs resulting from both experiments.

### Characterization of the vibrator

**Fig. 3:**
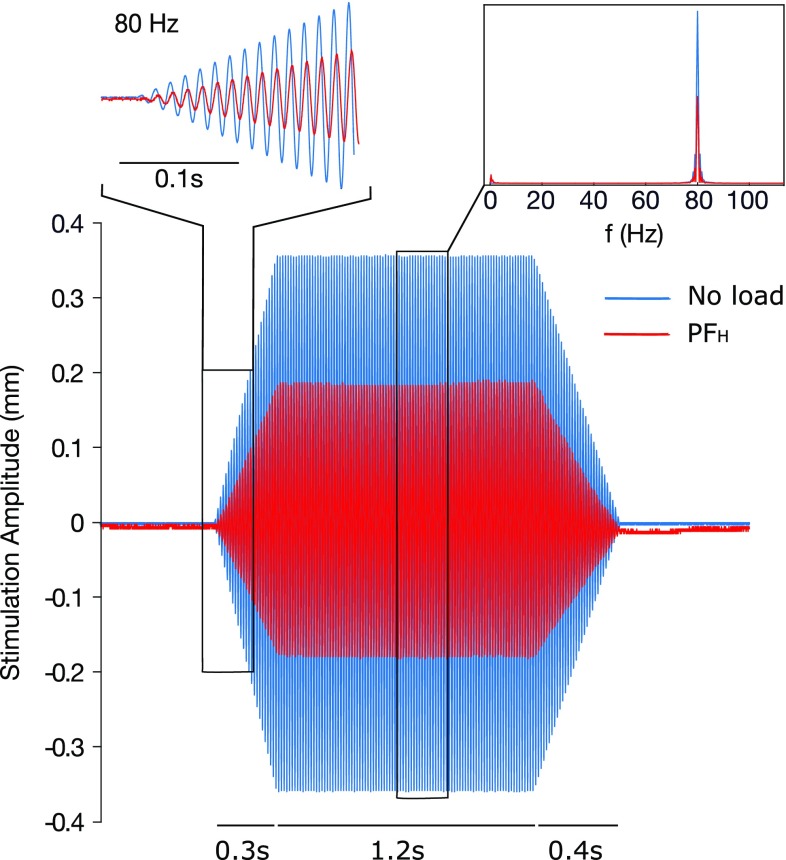
Sample trial for the PF_H_ level. During the experiment, the amplitude of the vibration was dampened by the skin depending on the applied PF. In particular, an average 55% reduction in the vibration amplitude was observed in the PF_H_ condition (red line) with respect to the no-load, ideal condition (blue line). The dampening had practically no effects in the frequency spectrum of the stimulations (top right box)

The measured attenuation of the vibration amplitude increased with the PF indicating that the compressed tissue becomes stiffer as the compression increases. In particular, the mean attenuation with respect to the no-load case proved equal to 14% (± 5% standard deviation), 27% (± 7%) and 55% (± 7%) in the PF_LL_, PF_L_ and PF_H_ conditions, respectively. Nonetheless, within each PF range, the attenuation demonstrated constant (7% maximum variability), implying the same experimental conditions throughout the study. Additionally, the frequency components of the stimulations were not altered by the variation of both amplitude and PF (Fig. 3). Indeed, the peak frequency of the stimulations throughout the whole experiment proved to be 79.9 ± 0.72.

### Training phase

Concerning the first experiment, during the training phase, three subjects immediately perceived the IoM, while the others needed three to five stimulation cycles to be able to perceive it. In addition, of the 16 subjects enrolled, three initially reported to perceive flexion of the elbow (instead of extension). In these cases, a reduction in the stimulation amplitude helped the participants to autonomously report the expected sensation. The other four participants did not complete the experiment because the vibratory stimulation did not elicit an IoM for at least one of the three stimulation points. For this reason, they were excluded from the data analysis, which thus only included 12 subjects. In the second experiment, as they had prior experience with IoM, all 12 subjects performed the experimental phase and were included in the data analysis. In this case, due to their prior experience, at most three stimulation cycles were needed to elicit the IoM.

### Experimental phase

The PF ranges were checked after each trial confirming the consistent application of the vibrator throughout the test. The Kolmogorov–Smirnov test demonstrated that the ATs were normally distributed (*p* > 0.05) for all the PF conditions.

The PF (mean ± standard deviation) measured, respectively, for the PF_LL_, PF_L_, and PF_H_ were 0.59 ± 0.08N, 1.58 ± 0.18N and 3.48 ± 0.19 N at the DT; 0.61 ± 0.061N, 1.68 ± 0.23N and 3.47 ± 0.24 N at the MB; 0.60 ± 0.09N, 1.66 ± 0.19N and 3.38 ± 0.20 N at the PT.

**Fig. 4:**
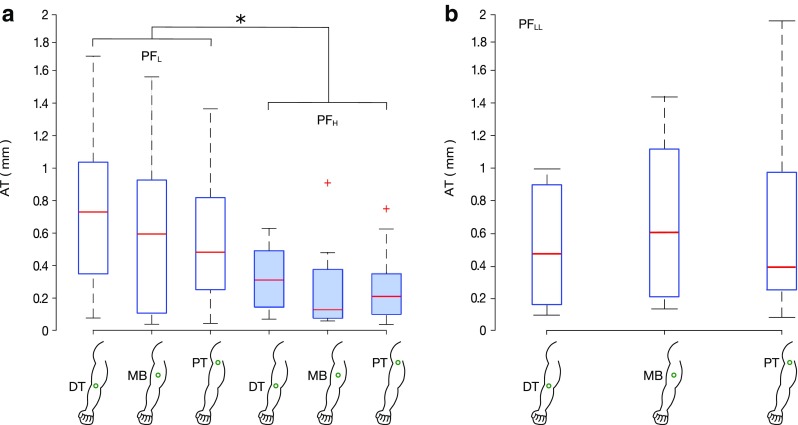
Minimum stimulation amplitude (amplitude threshold, AT) eliciting an illusion of movement. **a** In the first experiment, the AT was found to be affected by the PF range used (*F*(1,11) = 25.5, *p* = 0.0004). No statistical difference was found between different stimulation points (*F*(2,22) = 1.32, *p* = 0.28). PF_L_ (empty bars) and PF_H_ (filled bars) indicate the lower (1–2 N) and higher (3–4 N) preload force ranges, respectively. **b** In the second experiment, with a PF_LL_ range (0.5–0.75 N), the stimulation point did not influence the AT eliciting an IoM as well (*F*(2,22) = 1.160, *p* = 0.332). DT, MB and PT indicate the distal tendon, the muscle belly and the proximal tendon, respectively. *p < 0.05, +outliers defined as the points outside the interval [q1–1.5(q3 – q1), q3 + 1.5(q3 – q1)], where q1 and q3 are the 25th and 75th percentiles, respectively

In the first experiment, the AT proved inversely proportional to the PF, i.e. a larger PF led to smaller median stimulation amplitudes needed to elicit an IoM (Fig. 4a). In particular, the AT tripled (from 0.22 mm to 0.60 mm) from the PF_L_ to the PF_H_ condition (median across all stimulation points). On the other hand, changes were less evident between stimulation points (Fig. 4a): the median AT was 0.73, 0.60 and 0.48 mm with the PF_L_ range and 0.31, 0.13 and 0.21 mm with the PF_H_ range for the DT, MB, and PT, respectively. Results from the RM-ANOVA supported these observations, showing a significant effect of the PF range on the AT (*F*(1,11) = 25.5, *p* = 0.0004) and no effect of the stimulation point (*F*(2,22) = 1.32, *p* = 0.28). No significant interaction effects were found between the PF and the stimulation points (*F*(2,22) = 0.78, *p* = 0.472).

During the second experiment, the same test was repeated while setting an even lower PF range (0.5–0.75 N). In line with the previous experiment, no significant effect of the stimulation point was observed (RM-ANOVA, *F*(2,22) = 1.160, *p* = 0.332, Fig. 4b): the median AT was found to be 0.47 mm, 0.61 mm and 0.39 mm for the DT, MB and PT, respectively.

**Fig. 5:**
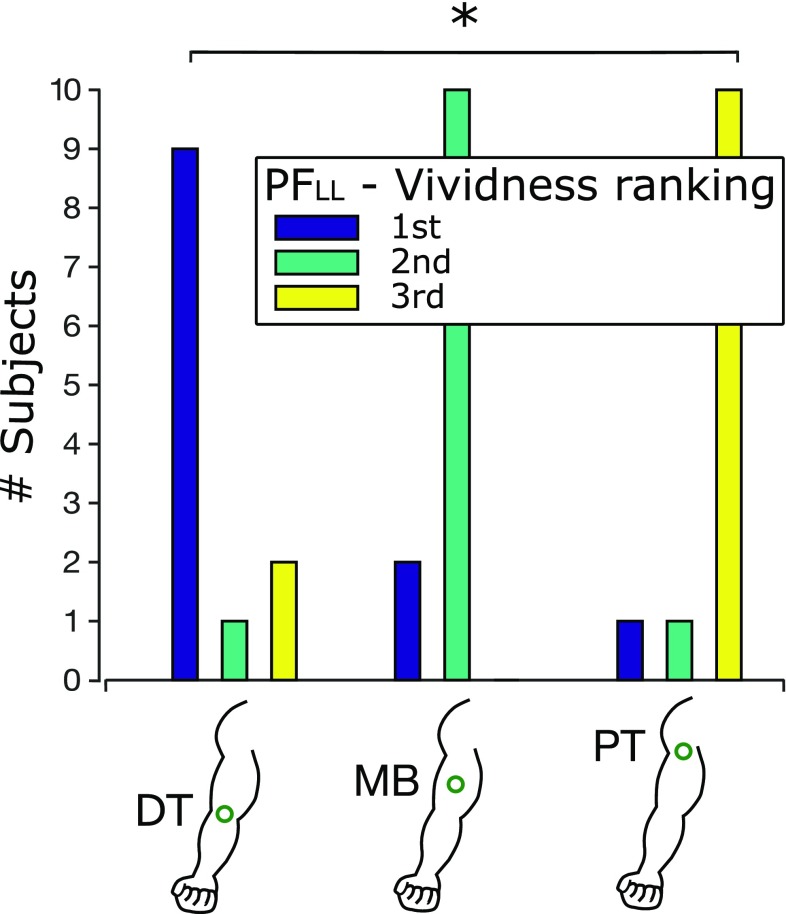
Vividness of the IoM (second experiment; PF_LL_=0.5–0.75 N). The subjects ranked the points of stimulation according to the strength of the IoM. Most subjects ranked the DT first (blue bar), i.e. the point with the strongest IoM, the MB second (green bar) and the PT third (yellow bar). A statistically different perception of the IoM emerged between the DT and the PT (*p* = 0.036). DT, MB and PT indicate the distal tendon, the muscle belly and the proximal tendon, respectively

Finally, 75% of the subjects reported to perceive an increased IoM vividness when the DT was stimulated, followed by MB and PT (Fig. 5). This was found statistically significant (Friedman test, *χ*^2^(2) = 10.66, *p* = 0.0048). The post hoc comparison (Wilcoxon signed-rank test with Bonferroni correction) showed a statistical difference only between the stimulation of DT and PT (*p* = 0.036). The difference between the DT and MB was found close to the statistical level (*p* = 0.063), whereas no difference was found between the MB and PT (*p* = 0.195).

## Discussion

The objective of the present study was to investigate the effects of the preload force and of the stimulation point on a vibration-induced IoM. Our experiments showed that (1) the PF affects the AT (in an inversely proportional way), (2) there is no evidence of an influence of the stimulation point on the AT, and (3) the vividness of the IoM changes depending on the stimulation point. Additionally, they confirmed that eliciting an IoM in a repeatable way is still a difficult task. Indeed, similar to previous reports (Schofield et al. 2015; Taylor et al. 2017), we found that (i) in four subjects we were unable to induce kinaesthetic sensations, (ii) a few subjects reported to perceive an IoM in the direction of flexion, and (iii) several trials were needed for some subjects to perceive the IoM.

As previously mentioned (Cordo et al. 1993) in the vibration-induced IoM, the interrelationship of the amplitude and the average force is complex. Most of the studies performed earlier did not specify the PF and even less attempted to regulate it (Gilhodes et al. 1986; Bergenheim et al. 2000; White and Proske 2009). Here, the design of a custom support guaranteed a precise positioning of the vibrator over the stimulated point (cf. Results). However, the inter-individual differences in anatomy and the tissue compliance prevented to set an exact value of the PF, especially with larger PFs. Hence, to standardize conditions, three force ranges were chosen based on the sparse information available from the literature. In particular, the two ranges PF_L_ and PF_H_ were similar to PFs used in previous studies (2.5–4 N in Schofield et al. 2015; 2.4 N in Tidoni et al. 2015; 2.4 and 4.2 N in Fusco et al. 2016), whereas the separation between the ranges was chosen considering that small differences in the PF (in the order of 0.5-1N) are known to be enough to affect the responsiveness of the muscle spindle afferents (Cordo et al. 1993).

We used stimulations having a relatively short vibration time (2 s) when compared to the literature (Gilhodes et al. 1986; Cordo et al. 2005; Fuentes et al. 2012; Tidoni et al. 2015). In fact, there is a lack consensus about the duration of the vibration required to elicit an IoM, as previous studies reported durations ranging from 1 to 30 s. The studies on the IoM latency time by Lackner and Taublieb (Lackner and Taublieb 1984) and by Goodwin et al. (Goodwin et al. 1972b) taken together suggest that the stimulation frequency plays an important role in how quickly the perceptual illusion arises. Close to the frequency of our interest, Goodwin et al. observed latencies for the illusion of less than 1 s, and as such, we chose 2 s for our experimental protocols. Nonetheless, the relatively short duration may explain why one-fourth of naïve participants felt no illusion, in the first experiment.

Our outcomes were based on established psychophysical methods, namely the stochastic approximation staircase procedure. In particular, an accelerated stochastic approximation method was implemented (Faes et al. 2007). The advantage of this procedure is the automatic adjustment of the stimulation step size depending on the subject’s response and on the number of reversals (Eq. 1). This tailored the task difficulty to the subjective performance, making the test less vulnerable to ceiling or flooring effects, and keeping the difficulty consistent across all experimental conditions (Clemente et al. 2017).

The ATs we found in the six experimental conditions ranged between 0.1 and 0.8 mm. To the best of our knowledge, this was not tested or reported earlier, making it difficult to validate our results. The closest comparison was the study by Fallon and colleagues (Fallon and Macefield 2007) that investigated the minimum stimulation amplitude needed to activate spindle afferents. By vibrating the distal tendons of foot and ankle muscles using 80 Hz sinusoidal vibrations, they found an AT of 0.17 mm. Although they stimulated completely different body sites, their results closely match ours.

Furthermore, we found that the PF affected the AT (Fig. 4) similarly for all the three stimulation points, as indicated by the absence of interaction effects between these factors. In particular, the AT was lower with a larger PF. These findings actually corroborate previous studies by Fusco and colleagues (Fusco et al. 2016) who determined that by increasing the PF, the vividness, the duration and the range of the IoM improved. Also Cordo and his colleagues (Cordo et al. 1993) found a similar effect of the PF. In particular, they reported a positive correlation between the PF and the discharge of the muscle spindle afferents: increments in the PF similar to the ones we tested (1–2 N) tripled the discharge of the spindle afferents. This matches closely with our result where increasing the PF led to a decrease of the AT by a factor of three on average (from 0.6 to 0.2 mm). According to this, we expected the highest ATs from the lowest PF range used in the last experiment. However, this was not the case as the median AT in the PF_LL_ case proved overlapped with that in the PF_L_ case. The apparent mismatch could be explained by two reasons. First, it is likely that such small forces fall within a poor sensitivity range of the sensory system involved and thus affect the AT in a similar way. In other words, the PFs involved in the PF_L_ and PF_LL_ ranges (which are very similar) seem to be too small (sub-threshold) to produce an appreciable effect. Second, in the second experiment (where the PF_LL_ was used), the subjects had prior experience with the illusion. Indeed, albeit the IoM of experienced and inexperienced subjects was never thoroughly compared, it was already suggested that earlier experience may increase the ability to perceive the illusion (Taylor et al. 2017), and thus reduce the AT.

This is the first study that investigates and reports that the AT for eliciting an IoM does not significantly change among three stimulation points of the same muscle (distal tendon, muscle belly and proximal tendon) (Fig. 4). This result emerged from both the first and second experiments, i.e. for all PF ranges tested. This “equivalence” of the stimulation points was only suggested by Burke and colleagues (Burke et al. 1976), who reported that it is possible to obtain an optimal spindle activation if the vibrator is accurately applied to the muscle tendon or to the muscle belly in the vicinity of the receptors. Nonetheless, this equivalent AT across stimulation points seems to contrast with the qualitative indications reported by the subjects. They described stronger sensations when the DT was stimulated (Fig. 5) reinforcing the preliminary observations from our pilot studies which encouraged us to start the first experiment by stimulating the DT. Previous literature which supported this idea hypothesized that stimulating the tendons elicits a longitudinal stretch of the muscle fibres which promotes a better transmission of the vibration to the muscles spindles (Taylor et al. 2017). However, as we did not find any evidence of a change in the AT among stimulation points, we argue that the muscle spindles are stimulated similarly in all conditions. We hypothesize two plausible explanations for the stronger illusion perceived at the DT: (1) a combined effect of different types of mechanoreceptors and (2) the subjective way of evaluating the IoM by the subjects. For the first point, it is indeed known that skin stretch close to the joint can generate an IoM (Edin and Johansson 1995) and that when this stretch is combined with vibration, the perceived IoM becomes stronger (Collins et al. 2005). We argue that the consistency of AT along the muscle suggests that for small vibratory stimulations (i.e. close to threshold levels, AT), the activation of the muscle spindles (which are stimulated independently from the position of the probe (Roll et al. 1989; Marasco et al. 2017)), is the only responsible for eliciting (or not) the IoM, whereas skin stretch close to the joint only modifies the qualities associated with the sensation (i.e. the speed and amplitude of the illusory movement). This is supported by previous research, which reported that vibration has a much stronger effect in eliciting the IoM than skin stretch at the elbow (Collins et al. 2005). In addition, it is known that supplementary information from other sensory channels can strongly modulate a perceived sensation (e.g. in the parchment-skin illusion, alteration in the sound produced by two rubbing hands changes their perceived roughness (Jousmäki and Hari 1998)). However, additional studies are needed to draw stronger conclusions. For the second point, the subjects might have evaluated the vividness of the IoM actually taking as a reference a particular feature of the illusion such as the range of motion, the velocity of the movement or the after effect. For each subject, these features might change between and among the different stimulated points, unconsciously affecting the rating of the illusion between experimental conditions. A more objective evaluation of the illusion, e.g. through the replication of the perceived movement using the contralateral body part, will be introduced in future works to confirm this hypothesis.

It should also be noted that there was a substantial difference between the two experiments. In the first one, the stimulation order of the three points was not randomized (the DT was always stimulated first), while it was randomized in the second. Although this choice might be seen as a limitation of the first experiment, it was dictated by the need to reduce the duration of the procedure, avoiding excessive cognitive fatigue in the subjects. In particular, the training session could be considerably shortened because, as we had learned from pilot experiments, the participants (without previous experience of the IoM) perceived more easily the IoM when the DT was stimulated. The second experiment, where the points were randomized, yielded similar results (i.e. no effect of the stimulation point on the AT). This validates the results from the first experiment, suggesting that in our setup no particular learning effects were introduced.

Based on our experience and results, the DT proved the best point to stimulate to elicit a strong IoM. It seems both more practical (simpler localization of the tendon) and more efficient (increased vividness) than the other points. Applying a large PF also promotes the illusion. However, we successfully elicited an IoM in all experimental conditions (i.e. for all three stimulation points and three PF ranges): our outcomes suggest to set the stimulation amplitude to 0.80 mm or more in the case of a small PF (i.e. less than 2N) and to at least 0.4 mm with larger PFs (i.e. more than 4 N). We invite further studies to investigate whether these results can be generalised to other muscles.

Amplitude reduction has been shown to correlate with reductions in the strength of illusory sensations, as well as the range and velocity of perceived motion (Schofield et al. 2015). This work complements these studies albeit marginally contributes to the debate about the quality and strength of the perceived sensations. Indeed, one of the limits of this study was the lack of assessment of the correlation between the AT and the qualities/features of the perceived illusion. Further studies are necessary to integrate this analysis with multiple PF ranges to optimize the selection of the vibration parameters that can effectively restore kinaesthetic sensations in clinical applications.

Nonetheless, the results from our work have a potential impact when inducing the IoM in a number of healthy or sensorimotor impaired populations (Ribot-Ciscar et al. 2003; Conrad et al. 2011; Marasco et al. 2018). The evidence that it is possible to elicit an IoM by vibrating different muscle areas is particularly interesting in the field of limb prosthetics. Indeed, it suggests that kinaesthetic sensations could be restored for different amputation levels, as long as any part of the target muscle is still available.

To conclude, several previous studies did not report if they controlled important parameters such as the preload force and the vibration amplitude (Gilhodes et al. 1986; Naito et al. 1999; Collins et al. 2000; Seizova-Cajic et al. 2007). Our outcomes instead suggest that a complete control over the stimulation parameters is crucial to compare results among participants and to study how the nervous system uses sensory feedback to monitor and control body movements.

## Abbreviations

PF: Preload force
PF_L_: Low preload force (1–2 N)
PF_H_: High preload force (3–4 N)
PF_LL_: Very low preload force (0.5–0.75 N)
AT: Amplitude threshold
PT: Proximal tendon
MB: Muscle belly
DT: Distal tendon
IoM: Illusion of movement
SAS: Stochastic approximation staircase

## References

[CR1] Albert F, Bergenheim M, Ribot-Ciscar E, Roll JP (2006). The Ia afferent feedback of a given movement evokes the illusion of the same movement when returned to the subject via muscle tendon vibration. Exp Brain Res.

[CR2] Ansems GE, Allen TJ, Proske U (2006). Position sense at the human forearm in the horizontal plane during loading and vibration of elbow muscles. J Physiol.

[CR3] Bergenheim M, Ribot-Ciscar E, Roll JP (2000). Proprioceptive population coding of two-dimensional limb movements in humans: I. Muscle spindle feedback during spatially oriented movements. Exp Brain Res.

[CR4] Brown MC, Engberg I, Matthews PBC (1967). The relative sensitivity to vibration of muscle receptors of the cat. J Physiol.

[CR5] Burke D, Hagbarth KE, Lofstedt L, Wallin BG (1976). The responses of human muscle spindle endings to vibration during isometric contraction. J Physiol.

[CR6] Calvin-Figuière S, Romaiguère P, Gilhodes JC, Roll JP (1999). Antagonist motor responses correlate with kinesthetic illusions induced by tendon vibration. Exp Brain Res.

[CR7] Clemente F, Håkansson B, Cipriani C (2017). Touch and hearing mediate osseoperception. Sci Rep.

[CR8] Collins DF, Refshauge KM, Gandevia SC (2000). Sensory integration in the perception of movements at the human metacarpophalangeal joint. J Physiol.

[CR9] Collins DF, Refshauge KM, Todd G, Gandevia SC (2005). Cutaneous receptors contribute to kinesthesia at the index finger, elbow, and knee. J Neurophysiol.

[CR10] Conrad MO, Scheidt RA, Schmit BD (2011). Effects of wrist tendon vibration on targeted upper-arm movements in poststroke hemiparesis. Neurorehabil Neural Repair.

[CR11] Cordo PJ, Gandevia SC, Hales JP (1993). Force and displacement-controlled tendon vibration in humans. Electroencephalogr Clin Neurophysiol.

[CR12] Cordo PJ, Gurfinkel VS, Brumagne S, Flores-Vieira C (2005). Effect of slow, small movement on the vibration-evoked kinesthetic illusion. Exp Brain Res.

[CR13] Craske B (1977). Perception of impossible limb position induced by tendon vibration. Science.

[CR14] Edin B, Johansson N (1995). Skin strain patters provide kinaesthetic information to the human central nervous system. J Physiol.

[CR15] Faes L, Nollo G, Ravelli F (2007). Small-sample characterization of stochastic approximation staircases in forced-choice adaptive threshold estimation. Percept Psychophys.

[CR16] Fallon JB, Macefield VG (2007). Vibration sensitivity of human muscle spindles and Golgi tendon organs. Muscle Nerve.

[CR17] Fuentes CT, Gomi H, Haggard P (2012). Temporal features of human tendon vibration illusions. Eur J Neurosci.

[CR18] Fusco G, Tidoni E, Barone N (2016). Illusion of arm movement evoked by tendon vibration in patients with spinal cord injury. Restor Neurol Neurosci.

[CR19] Gilhodes JC, Roll JP, Tardy-Gervet MF (1986). Perceptual and motor effects of agonist-antagonist muscle vibration in man. Exp Brain Res.

[CR20] Goodwin GM, McCloskey DI, Matthews PBC (1972). Proprioceptive illusions induced by muscle vibration: contribution by muscle spindles to perception?. Science.

[CR21] Goodwin GM, McCloskey DI, Matthews PBC (1972). The contribution of muscle afferents to kinaesthesia shown by vibration induced illusions of movement and by the effects of paralysing joint afferents. Brain.

[CR22] Gordon J, Ghilardi MF, Ghez C (1995). Impairments of reaching movements in patients without proprioception. I. Spatial errors. J Neurophysiol.

[CR23] Guerraz M, Provost S, Narison R (2012). Integration of visual and proprioceptive afferents in kinesthesia. Neuroscience.

[CR24] Jones LA (1988). Motor illusions: what do they reveal about proprioception?. Psychol Bull.

[CR25] Jousmäki V, Hari R (1998). Parchment-skin illusion: sound-biased touch. Curr Biol.

[CR26] Kandel E (2013). Principles of neural science.

[CR27] Kesten H (1958). Accelerated Stochastic Approximation. Ann Math Stat.

[CR28] Kingdom F, Prins N (2010). Psychophysics: a practical introduction, 1st edn.

[CR29] Kito T, Hashimoto T, Yoneda T (2006). Sensory processing during kinesthetic aftereffect following illusory hand movement elicited by tendon vibration. Brain Res.

[CR30] Lackner JR, Taublieb AB (1984). Influence of vision on vibration-induced illusions of limb movement. Exp Neurol.

[CR31] Marasco PD, Bourbeau DJ, Shell CE (2017). The neural response properties and cortical organization of a rapidly adapting muscle sensory group response that overlaps with the frequencies that elicit the kinesthetic illusion. PLoS One.

[CR32] Marasco PD, Hebert JS, Sensinger JW (2018). Illusory movement perception improves motor control for prosthetic hands. Sci Transl Med.

[CR33] Naito E, Ehrsson HH, Geyer S (1999). Illusory arm movements activate cortical motor areas: a positron emission tomography study. J Neurosci.

[CR34] Naito E, Morita T, Amemiya K (2016). Body representations in the human brain revealed by kinesthetic illusions and their essential contributions to motor control and corporeal awareness. Neurosci Res.

[CR35] Ribot-Ciscar E, Butler JE, Thomas CK (2003). Facilitation of triceps brachii muscle contraction by tendon vibration after chronic cervical spinal cord injury. J Appl Physiol.

[CR36] Roll JP, Vedel JP (1982). Kinaesthetic role of muscle afferents in man, studied by tendon vibration and microneurography. Exp Brain Res.

[CR37] Roll JP, Vedel JP, Ribot E (1989). Alteration of proprioceptive messages induced by tendon vibration in man: a microneurographic study. Exp Brain Res.

[CR38] Schofield J, Dawsonb MR, Careya JP, Hebertb JS (2015). Characterizing the effects of amplitude, frequency and limb position on vibration induced movement illusions: implications in sensory-motor rehabilitation. Technol Heal Care.

[CR39] Seizova-Cajic T, Smith JL, Taylor JL, Gandevia SC (2007). Proprioceptive movement illusions due to prolonged stimulation: reversals and aftereffects. PLoS One.

[CR40] Sherrington C (1906). The integrative action of the nervous system.

[CR41] Taylor MW, Taylor JL, Seizova-Cajic T (2017). Muscle vibration-induced illusion: review of contributing factors, taxonomy of illusion and user’s guide. Multisens Res.

[CR42] Tidoni E, Fusco G, Leonardis D (2015). Illusory movements induced by tendon vibration in right- and left-handed people. Exp Brain Res.

[CR43] White O, Proske U (2009). Illusions of forearm displacement during vibration of elbow muscles in humans. Exp Brain Res.

